# Algunos dilemas éticos presentes y futuros ante los avances en fecundación in vitro

**DOI:** 10.18294/sc.2023.4462

**Published:** 2023-11-16

**Authors:** Marta Reguera Cabezas

**Affiliations:** 1 Bióloga, Magíster en Genética y Reproducción Humana Asistida, Unidad de Reproducción Asistida, miembro del Comité de Ética Asistencial, Hospital Universitario Marqués de Valdecilla, Cantabria, España. marthareguera@yahoo.es Hospital Universitario Marqués de Valdecilla Unidad de Reproducción Asistida Comité de Ética Asistencial Hospital Universitario Marqués de Valdecilla Cantabria España marthareguera@yahoo.es

**Keywords:** Fecundación in Vitro, Edición Genética, Ectogénesis, Técnicas Reproductivas, Bioética, Fertilization in Vitro, Gene Editing, Ectogenesis, Reproductive Techniques, Bioethics

## Abstract

El creciente campo de la reproducción humana asistida ha alcanzado hitos inimaginables. Su continuo desarrollo y las innovaciones que genera, en ocasiones, plantean dilemas tanto éticos como jurídicos. El presente ensayo trata de exponer los cambios progresivos que se están viviendo en el ámbito del origen de la vida debido al desarrollo de nuevas opciones y estrategias en reproducción humana asistida. En primer lugar, se realiza una reflexión interdisciplinar desde la ciencia, la ética y el derecho, sobre la naturaleza humana y los cambios a los que la sociedad se enfrenta, en particular, desde la perspectiva española. En segundo lugar, recoge una breve aproximación en torno a las técnicas biomédicas presentes o futuras en el campo de la reproducción humana. Concluye sobre la necesidad de reflexionar ante el vertiginoso avance de la ciencia en materia de reproducción humana asistida.

## Abstract (en)

The growing field of assisted human reproduction has achieved unimaginable milestones. Its continuous development and the innovations it generates at times pose both ethical and legal dilemmas. This essay aims to elucidate the progressive changes occurring in the realm of the origin of life due to the development of new options and strategies in assisted human reproduction. First, it constructs an interdisciplinary reflection on human nature and the changes society faces from the perspectives of science, ethics, and law, particularly from the perspective of Spain. Second, it provides a brief overview of current or future biomedical techniques in the field of human reproduction. It concludes with a discussion of the need to reflect on the rapid advancement of science in assisted human reproduction.

## INTERACCIÓN DE LA CIENCIA Y LA NATURALEZA HUMANA

La reflexión bioética y jurídica en torno a la trasformación del concepto de naturaleza humana está de plena actualidad debido al avance científico en la biología humana. No estamos tan lejos de aquellas reflexiones de Ortega y Gasset, abogando por el estudio de la naturaleza humana. Parece que el tiempo no ha conseguido doblegar las intensas reflexiones sobre la naturaleza del hombre. En lugar de ello, podríamos atrevernos a decir que los avances biotecnológicos, instaurados y futuros, no han hecho más que avivar el debate sobre el estudio de la naturaleza humana, su diversidad, su enfermedad, o su genoma, entre otros.

Sería aventurado cuestionar hasta cuándo estarán vigentes las teorías darwinistas, pues se abren ante nosotros posibilidades biotecnológicas de la mano de la fecundación in vitro y la edición genética que acelerarían el proceso evolutivo a través de la intervención del ser humano. Esto genera un complejo debate sobre la pertinencia, los riesgos y los límites de las intervenciones humanas en concreto, ante la edición genética en la línea germinal^1^. Para algunos, lejos de plantearse los riesgos e incertidumbres, simplifican su reflexión, considerando que la parte más compleja de la revolución de dichos avances biomédicos es que “no puedes comprarla a plazos”, es decir, no puedes escoger solo aquellos aspectos que más te interesen o agraden, tal y como plantea Peter Singer en su obra^2^. 

Las posturas en relación con las intervenciones en el ser humano son contrapuestas. Así, nos podemos encontrar en la literatura obras con extensos argumentos defensores como los de Savulescu^3^ y también detractores de la intervención genética en embriones humanos, para los cuales, la naturaleza y la complejidad de esos nuevos avances científicos son una actitud aventurada y arriesgada de la ciencia y la biotecnología. La reproducción sexual sigue marcando el camino de la evolución como mecanismo enriquecedor de nuestra diversidad. Algunas posiciones expresan que la población no dejará de reproducirse de forma natural siempre que le sea posible. Sin embargo, para otros esto dependerá del costo-beneficio, es decir, de las opciones que puedan aportarles las nuevas biotecnologías reproductivas no solo en salud^2^. Es otras palabras, las libertades reproductivas^4^, se transformarían en libertades que tienen un alto precio^5^. Esto ha repercutido en los informes de varios organismos y comités europeos e internacionales, que centraron sus miradas en minimizar el impacto antropológico y social que las biotecnologías reproductivas puedan generar^6^.

El actual estado de desarrollo de la medicina reproductiva y una regulación jurídica muy favorable en España hacen que se pueda ofrecer un gran abanico de opciones técnicas y terapéuticas, ante las cuales, se deben debatir y consensuar las delimitaciones; retos que serán planteados y abordados de forma diferente según las tradiciones, influencias religiosas y normativas internas de cada país. No obstante, en cualquier latitud queda patente la necesidad de reflexionar sobre estas cuestiones^7^, pues de no existir ciertos límites jurídicos ante nuevas técnicas, en su indicación o en su alcance, pueden llegar, inevitablemente, a provocar grandes desigualdades e injusticias en la sociedad^8^, incluso riesgos para el ser humano.

El presente ensayo se desarrolla bajo una metodología de investigación bibliográfica y reflexiva, la cual tiene como objetivo proporcionar un marco del estado actual y los retos del tema abordado y reflexionar sobre los resultados, retos, así como identificar posibles áreas de mejora o de futuras investigaciones.

## AVANCES BIOTECNOLÓGICOS EN MEDICINA REPRODUCTIVA Y GENÉTICA

Los avances biomédicos del presente siglo suponen un reto para la bioética. En el ámbito de la fecundación in vitro posiblemente se vuelva más necesaria que nunca, pues el inicio de la vida comienza a exponerse a situaciones que ofrecen grandes posibilidades a la vez que riesgos desconocidos^9^.

Numerosos expertos del ámbito de la ética han abordado los grandes dilemas que aún hoy siguen presentes, como la investigación con embriones humanos, el diagnóstico genético preimplantacional (DGP) desde la perspectiva eugenésica, al altruismo de las donaciones de gametos o el turismo reproductivo.

No obstante, en los últimos años en el ámbito de la medicina reproductiva hemos vivido un giro propiciado en gran medida por tres factores: el incesante retraso en la edad de maternidad, el paso de la medicina personalizada a los tratamientos de fertilidad como objeto de consumo y la industrialización de la medicina reproductiva. Estos cambios han traído consigo prácticas y efectos que deben de analizarse pues pueden implicar consecuencias éticas que condicionarán hacia dónde se dirija la practica biomédica en el futuro. A pesar de los avances logrados en las últimas cuatro décadas en reproducción asistida, los resultados parecen haber progresado a un ritmo menor, incluso en tratamientos de fecundación in vitro sin contribución de gametos donados parece que los resultados han disminuido discretamente^10^.

Pretendemos hablar de avances reproductivos, para lo cual nos vamos a guiar de un referente en la bioética europea y mundial, el *Nuffield Council on Bioethics* del Reino Unido*,* uno de los organismos que promueven y velan por el seguimiento de las directrices éticas en biomedicina. Una institución que genera conocimientos y actualiza de forma continua sus contenidos y los temas de interés para su análisis y divulgación a la sociedad. El *Nuffield Council on Bioethics* nos presenta los modernos desafíos existentes en la sociedad para la bioética, y los clasifica en las siguientes categorías^11^: *“beginning of life; health and society; data and technology; animal, foods and environment; research on ethics”.*

El presente trabajo se centra en la primera categoría: “*beginnig of life”* en la que se enumeran algunos de los dilemas éticos que se plantean con la incorporación de las nuevas biotecnologías al inicio de la vida^11^. Desde el descubrimiento de la fecundación in vitro, este campo no ha dejado de expandirse. Tras más de cuarenta años, la sociedad ha normalizado su convivencia con los tratamientos de fecundación in vitro y los llamados bebes probeta. Incluso parece haber superado la controversia eugenésica que siempre ha planeado sobre el diagnóstico genético preimplantacional y se ha regularizado la selección de embriones histocompatibles. Nos atreveríamos a denominar todos estos avances como los viejos retos de la fecundación in vitro^12^, ¿qué nos deparan los nuevos retos en la formación y desarrollo de embriones in vitro?^13^.

En el transcurso del presente siglo nos enfrentaremos a retos de mayor complejidad de forma global. La pretensión de este trabajo es realizar una breve aproximación al estado actual de estos retos e introducir algunos de los dilemas ético-jurídicos que se plantean en España.

## RETOS ACTUALES

### La congelación de óvulos como estrategia ante una natalidad en recesión

Como afirma la Organización de Naciones Unidas (ONU) nos encontramos ante una situación con una fecundidad por debajo del nivel de reemplazo que consolida el cambio social instaurado a nivel internacional. La mayoría de los expertos comparten cómo el creciente retraso en la edad de maternidad es una respuesta al condicionante socioeconómico y profesional de la mujer. “La fecundidad por debajo del nivel de reemplazo probablemente será la norma global en las próximas décadas”^14^. Ante tal perspectiva, la mayoría de los estados no han planteado aún estrategias con la finalidad de minimizar dicha tendencia. El retraso de la edad de maternidad supone la visualización de un problema coyuntural mayor derivado de la difícil conciliación laboral y familiar de la mujer. 

En la última década se ha introducido como medida biomédica la vitrificación de los óvulos “por motivos sociales”. Esta técnica permite a mujeres jóvenes, que desean retrasar voluntariamente su fertilidad, mantener sus óvulos congelados. Sin embargo, a pesar de la efectividad de la vitrificación, las técnicas reproductivas no permiten lograr gestaciones a cualquier edad, ni en todos los casos^15^. 

La preservación social de la fertilidad pasó de ser un tratamiento médico a un fenómeno social; sin embargo, su auge inicial se ha visto moderado. Es una técnica que conlleva algunos dilemas bioéticos: la preservación social de óvulos puede condicionar la autonomía reproductiva de la mujer, supeditándola a otros intereses, como los empresariales, cuando son las multinacionales quienes fomentan esta práctica entre sus empleadas^16^^,^^17^^,^^18^^)”^. Es una estrategia con la finalidad de retrasar planificadamente la etapa reproductiva^19^, incrementado con ello el envejecimiento reproductivo y la edad a la que las mujeres acuden a tratamientos de fecundación in vitro^20^, lo cual está directamente asociado a un aumento de las complicaciones gestacionales y neonatales^21^^,^^22^. La vitrificación de óvulos, aún siendo una técncia segura y eficaz, no es garantía de una posible maternidad^23^, muchas de esas mujeres no conseguirán un embarazo con los ovocitos vitrificados^24^. Su uso social requiere de un consentimiento informado complejo que proporcione a las pacientes datos correctos sobre las opciones futuras^25^. 

Las soluciones para revertir los actules indices de natalidad, posiblemente tengan que abordarse desde varias perspectivas, por un lado, la biomédica y, por otro, de forma más integral, a través del compromiso de toda la sociedad.

### Los embriones sobrantes sin destino: los límites de la investigación con embriones humanos

En el año 1998, en España, la Comisión Nacional de Reproducción Humana Asistida (CNRHA) puso de manifiesto un problema que sigue siendo actual: la incesante acumulación de embriones sin destino sobrantes de los tratamientos con técnicas de reproducción humana asistida (TRHA). Esta situación no es exclusiva de España, sino que otros autores la han investigado en el entorno europeo, EEUU o Latinoamérica, en particular, en Argentina^26^^,^^27^^,^^28^^,^^29^^,^^30^^,^^31^.

La compleja y diversa realidad social nos muestra que los destinos para los embriones criopreservados previstos en la normativa española, concretamente en la Ley 14/2006, del 26 de mayo, de técnicas de reproducción humana asistida, no deja grandes márgenes de actuación. Existe una tendencia al abandono de los embriones sobrantes por parte de los pacientes una vez finalizado su proceso reproductivo^32^^,^^33^. Entre las opciones previstas por la legislación, la destrucción embrionaria, contemplada siempre como la última opción y en un contexto muy delimitado, se convierte paradójicamente en el destino más demandado para los embriones frente al resto de alternativas legales^33^.

Otro de los destinos que contempla la normativa española para los embriones sobrantes es la investigación. Según la Ley 14/2006 y la Ley 14/2007 de Investigación biomédica, la pareja o mujer progenitora puede, previa firma del consentimiento informado, realizar la donación de los embriones sobrantes a un proyecto de investigación concreto, autorizado por la Comisión Nacional de Reproducción Humana Asistida o por la Comisión de Garantías para la Donación, Utilización de Células y Tejidos Humanos, limitando la investigación a un periodo que no supere los 14 días de desarrollo embrionario contado desde la fecundación^34^. 

A finales de la década de 1970, la fecundación in vitro comenzaba su desarrollo clínico y expansión internacional, generando con ello la incertidumbre sobre los posibles riegos desconocidos para la descendencia y sus, por entonces, limitados resultados. La experimentación en este campo se volvía esencial para el perfeccionamiento técnico. Sin embargo, ante la posible explosión de la experimentación con embriones humanos sin control, se determinó la pertinencia de establecer un límite en el cual quedase amparada la fecundación in vitro y el embrión humano. Desde que en 1984 se publicó el *Report of the Committee of Inquiry Into Human Fertilisation and Embryology*^35^*,* conocido como el Informe Warnock, el límite de 14 días para el desarrollo embrionario in vitro fue incorporado a numerosas normativas internacionales, por ejemplo, en EEUU, Canadá, Reino Unido, Suecia, España, Dinamarca, Australia, China, entre otros, mientras que países como Alemania e Italia no permiten la investigación con embriones humanos.

En la actualidad dicho límite sigue vigente; sin embargo, no se encuentran argumentos sólidos que lo avalen. Numerosas publicaciones científicas cuestionan si el límite de 14 días debe ser modificado o no^36^^,^^37^^,^^38^^,^^39^. Este debate se centra en la pertinencia de su ampliación y en cuáles serían los nuevos límites establecidos. Los científicos abogan por su extensión debido a los innumerables beneficios terapéuticos^40^ que se derivarían del estudio del desarrollo embrionario durante la embriogénesis temprana conocida como gastrulación^41^, entre el día 14 y la cuarta semana de desarrollo^37^, contribuyendo a una mejor comprensión de algunos casos de abortos espontáneos y malformaciones congénitas^42^. A pesar de que existen reticencias a abrir este debate, la legislación es una herramienta dinámica controlada por el hombre y orientada según las distintas sociedades^43^, por ello permitiría la revisión y actualización de este límite, lo cual parece necesario. 

El gran dilema ético sobre la investigación con embriones sigue condicionado por las diferentes posiciones sobre el inicio de la vida humana^44^. Seguimos sin tener una respuesta para una pregunta tan compleja como ¿cuál es el estatus ético y jurídico de un embrión humano?, pregunta que Morgan L, simplifica cuestionándose ¿si un embrión es o no persona? Esta autora expone un amplio argumento defendiendo que un embrión es mucho más que un término biológico o una etapa del desarrollo^45^. A lo expuesto anteriormente se suman pesamientos opuestos que avivan el debate, entre ellos la visión utilitarista de la investigación con embriones humanos^46^, la finalidad de la investigación, la seguridad de las intervenciones, o la capacidad para modificar el genoma embrionario^47^^,^^48^. 

En la actualidad, la investigación a través de la embriología sintética se ha convertido en la alternativa a la investigación con embriones humanos^49^. La derivación de células madre embrionarias humanas y de ratón ha brindado la oportunidad de trabajar con embrioides, estructuras formadas por ensamblajes multicelulares que se asemejan a embriones naturales, tanto en la tipología celular como en su organización tridimensional^50^. La mayor parte de la investigación se centra en la reconstitución parcial de partes embrionarias, independientemente de la etapa de desarrollo^51^. 

Actualmente, hay pocas regulaciones explícitas con respecto a los embrioides humanos^52^. Una cuestión controvertida es si los embrioides son más que un modelo de investigación y, en consecuencia, comparten el mismo estatus ético que un embrión humano. No obstante, ninguno de los modelos creados ha demostrado aún su capacidad para desarrollarse durante más de unos pocos días in vitro. Estrictamente hablando, solo los embriones humanos en el útero tendrían un potencial activo para convertirse en un ser humano^53^. 

Otra de las cuestiones es la investigación con modelos embrioides, las directrices de la Sociedad Internacional para la Investigación de Células Madre (ISSCR) de 2021 mantiene la prohibición sobre la investigación en torno a los 14 días de desarrollo basándose en un “amplio consenso internacional de que tales experimentos carecen de una justificación científica convincente, plantean preocupaciones éticas sustanciales y/o son ilegales en muchos países”^42^^,^^54^. Por ello, debemos discutir la posibilidad de crear embrioides humanos para investigación que intenten modelar el desarrollo integrado de un embrión antes y después de 14 días, en lugar de utilizar embriones humanos, pues en la actualidad, los modelos no integrados carecen de potencial de implantación^50^^,^^55^.

### El uso de complementos en los tratamientos de fecundación in vitro

En los últimos años, en el área de la medicina reproductiva ha proliferado un extenso número de técnicas complementarias a los tratamientos de fecundación in vitro con la finalidad de mejorar las tasas de recién nacido^47^. El uso de estas técnicas denominadas complementos, también conocidos por su denominación inglesa “*add-ons*”, se ha extendido rápidamente a nivel mundial, en ocasiones con una prematura introducción en la práctica clínica rutinaria sin contar con una evidencia clínica suficientemente lógica^56^^,^^57^. Sin embargo, es complejo determinar el grado de uso de estos complementos, pues no están recogidos en los datos de actividad registrados por los distintos gobiernos y sociedades científicas.

La atención ética a la práctica rutinaria de los complementos de fecundación in vitro por parte de organizaciones profesionales y comités de ética es limitada. El objeto de este apartado es proponer una reflexión sobre la incorporación rutinaria en los tratamientos de fecundación in vitro de técnicas que requieren de una mayor investigación y consolidación de su efectividad y seguridad. Vemos cómo algunos autores exponen que los datos relacionados con los resultados, efectividad, incertidumbres y efectos adversos presentes en el consentimiento informado también son limitados. Esta situación de escasa información condiciona la toma de decisiones respecto a su uso por parte de los pacientes. Estos complementos -como son el rascado endometrial, pegamento embrionario, esteroides para suprimir la inmunidad, la inyección fisiológica de espermatozoides (PICSI), el rejuvenecimiento ovárico- son intervenciones no esenciales, que se ofrecen a pacientes de fecundación in vitro buscando un rendimiento superior, una ventaja competitiva, o tal vez simplemente ofrecer las mismas prestaciones que otras clínicas^58^.

En la actualidad, la autorregulación de la medicina reproductiva junto con las fuerzas del mercado, parece representar el estándar para la introducción de las innovaciones de fecundación in vitro en diferentes puntos del mundo^46^^,^^47^. La regulación normativa de los complementos de fecundación in vitro es mínima o nula en la gran mayoría de países^59^ ^60^ ^61^. 

El único ejemplo al que podemos hacer referencia es el Reino Unido, donde la autoridad de regulación Human Fertilization & Embriology Authority (HFEA) tiene un poder limitado para controlar el uso de complementos^62^. Al considerar un nuevo tratamiento, la HFEA solo puede rechazarlo por motivos de seguridad. Llama la atención que la efectividad no es una consideración. Sin embargo, este organismo regulador ha emitido una declaración de consenso que describe varios principios de innovación responsable^63^. La HFEA proporciona información a los consumidores para que sean conscientes de que algunos de los complementos pueden no mejorar sus posibilidades reales de éxito, para lo cual ha establecido un sistema de señalización de la evidencia de estos complementos^64^.

Se ha argumentado que debería requerirse una revisión reglamentaria completa antes de la introducción de un nuevo tratamiento reproductivo, a menos que no haya más que problemas mínimos de seguridad en comparación con la norma actual, no haya riesgo de reducción de las tasas de nacidos vivos y no haya riesgos de daño social^65^. 

Uno de los dilemas éticos que plantean algunas de las nuevas técnicas complementarias a los tratamientos de fecundación in vitro es su rápida introducción al ámbito clínico, en ocasiones, sin una clara evidencia científica de su efectividad^66^. Se ha descrito un paradigma ideal para el desarrollo e introducción de nuevas técnicas embriológicas, comenzando con la investigación básica basada en hipótesis y pasando por distintas etapas de investigación en embriones humanos donados y ensayos clínicos de creciente magnitud y alcance, que culminan en una evaluación exhaustiva de las tecnologías sanitarias^67^.

El segundo dilema es la obtención del consentimiento informado, que exige la obligación de garantizar que los pacientes comprendan los riesgos y beneficios de proceder con una intervención, proporcionando información relevante, así como aclarando información incompleta o engañosa, y asegurando que los pacientes tomen sus decisiones^68^^,^^69^. Dado que gran parte de los complementos son intervenciones experimentales, los pacientes se enfrentan a tomar decisiones sin tener claros los beneficios^70^ ^71^.

El tercero de los dilemas éticos que nos plantea son las cargas emocionales y financieras de los pacientes al someterse a tratamientos de fertilidad, las cuales pueden ser demasiado elevadas. Respecto a las cargas emocionales, los pacientes pueden no estar en condiciones de tomar decisiones sobre qué procedimientos elegir o rechazar. Con relación a las financieras, la introducción de complementos supone un encarecimiento de los tratamientos de fecundación in vitro, situación que se ha visto acentuada por un cambio de modelo en la práctica clínica^10^^,^^18^^,^^55^.

### Trasplante uterino: una técnica experimental

El trasplante de útero (UTx), aún experimental, pero en el que están apoyadas grandes expectativas a futuro^72^, brinda la posibilidad de completar la maternidad con la experiencia vital de la gestación^73^. Posiblemente, la doctrina ética por excelencia en el marco de la donación de órganos sea el utilitarismo. La exigencia del beneficio del acto de la donación -salvar vidas, ganar años y mejorar la calidad de vida- podría conllevar la obligación moral de la donación de órganos cuando los costos personales son muy inferiores a los beneficios que dicha donación confiere. Sin embargo podría parecer, *a priori*, que el trasplante uterino no tiene esa finalidad al no tratarse de un órgano vital. Cabria recordar que, desde una perspectiva ética, asentada en el concepto de salud de la Organización Mundial de la Salud, que dice que la salud no solo consiste en curar enfermedades o en no padecer ninguna, sino en el concepto integral de bienestar, físico, mental y social, donde cabe incorporar los derechos reproductivos. De este modo, el trasplante uterino, al igual que las técnicas citadas anteriormente, no está exento de controversia por factores como son el trasplante de órganos no vitales, el uso de donantes vivas con un potencial desequilibrio en el riesgo-beneficio^74^, la transitoriedad del injerto para la mujer receptora, o la pérdida del injerto sin conseguir descendencia^75^. Además, implica otros condicionantes ético-jurídicos, como la generación de embriones sin garantías de uso, dificultades de acceso a la técnica derivada de los costos, el riesgo de mercantilización de los trasplantes de útero, el riesgo de comercialización de órganos no vitales. El trasplante de útero es, sin duda, un hito destinado a dar lugar a estos y otros dilemas bioéticos altamente complejos^76^^,^^77^^,^^78^.

## RETOS FUTUROS

### Reprogramación celular: obtención de gametos humanos

Los primeros estudios sobre líneas celulares pluripotentes en humanos supusieron un impulso a la investigación en reprogramación celular^79^. Sin embargo, en el campo de la gametogénesis sigue siendo altamente ineficiente. Las células germinales primordiales -*primordial germ cells* (PGC)- son células madre pluripotentes embrionarias tempranas, son la primera población de células germinales establecida durante el desarrollo embrionario temprano del nuevo individuo^80^, y parecen estar preprogramadas y diferenciarse fácilmente en gametos^81^. A partir de este modelo, se han utilizado varias opciones para obtener gametos, incluidas las células madre pluripotentes -células madre embrionarias (CE) e *induced pluripotent stem cell* (iPS)-, células madre gonadales -*spermatogonial stem cells* (SSC) o *oogonial stem cells* (OSC)-, médula ósea, células mesenquimales y piel fetal. 

Este campo plantea un gran desafío que incluye protocolos existentes ineficientes para la diferenciación, cambios epigenéticos y genéticos asociados con una extensa manipulación*in vitro*y también restricciones ético-regulatorias. 

En 2011, se publicó en*Cell*que es posible obtener crías vivas a partir de espermatozoides derivados de células madre pluripotentes (CE e iPS)^82^. En 2012, publicaron en *Science*, siguiendo una estrategia similar, la posibilidad de obtener ovocitos derivados de CE o iPS^83^, además de la diferenciación de PGC (SSC y OSC) en gametos. Hermann *et al*. obtuvieron espermatozoides funcionales después del trasplante SSC autólogo en primates no humanos y posteriormente fueron utilizados para fecundación in vitro^84^. Por su parte, el grupo de Tilly ha hecho contribuciones significativas al campo de las OSC, demostrando la efectividad de su trasplante y diferenciación de ovocitos funcionales con el nacimiento de crías de ratón^85^^,^^86^.

En 2014, un nuevo estudio aportaba indicios prometedores en biología reproductiva. La publicación avanzaba la posibilidad de reprogramar células somáticas a células madre con la finalidad de dirigir su diferenciación hacia diferentes líneas celulares, en este estudio en particular, hacia células germinales^87^^,^^88^. 

Desde entonces, se han abierto nuevas posibilidades en el campo de la reprogramación celular^89^. Uno de esos campos es la obtención de gametos humanos a partir de células indiferenciadas. Su finalidad no solo es demostrar que es posible desarrollar óvulos y espermatozoides, sino que es posible conseguir descendencia con ellos^90^, aunque en seres humanos todavía hay muy poca evidencia para sugerir un posible uso clínico^91^. De lograrse, podría tener consecuencias impredecibles, el suministro de gametos sería potencialmente ilimitado^83^ y las cuestiones éticas subyacentes a esta opción se vuelven también ilimitadas.

Los gametos derivados de células madre pluripotentes podrían proporcionar opciones reproductivas potenciales a individuos con infertilidad secundaria a lesiones, exposición a tóxicos o tratamientos inmunosupresores, en casos con insuficiencia gonadal debido a insuficiencia ovárica prematura o azoospermia, envejecimiento reproductivo y casos idiopáticos de mala calidad de gametos y fracaso de la fecundación in vitro. Estos gametos artificiales derivados de células madre también pueden servir como un sistema modelo invaluable para estudiar la programación genética y epigenética del desarrollo de células germinales in vivo y también ayudar a obtener mejores conocimientos sobre las causas de los casos idiopáticos de infertilidad^81^.

Las implicaciones de los avances científicos rara vez se afrontan jurídicamente antes de su realización. Algunos expertos en bioética piden que se lleven a cabo “deliberaciones reflexivas ante-hoc sobre la perspectiva de los gametos humanos derivados de células madre, con la vista puesta en minimizar las posibles imposiciones normativas o estatutarias post hoc”^92^.

### La edición genética de embriones humanos

Pocos cuestionan el enorme potencial de la técnica en la mejora de la salud y el tratamiento de diversas enfermedades. La posibilidad de controlar y manipular la información genética de nuestra especie genera en la comunidad científica una fascinación por el desarrollo de la tecnología de repeticiones palindrómicas cortas agrupadas y regularmente interespaciadas o *clustered regularly interspaced short palindromic repeats* (CRISPR). Concretamente, “los límites de CRISPR llegan hasta donde llegue la imaginación de los científicos”^93^. Si bien nos encontramos en fases muy tempranas de la investigación en el campo de la edición genética de la línea germinal humana (EGLGH), los riesgos reales asociados a su aplicación deben medirse con sumo detalle, ante un avance en la investigación que impresiona incontrolable^94^.

El Convenio de Oviedo de 1997 excluye firmemente cualquier intervención genética intencional sobre el material reproductivo, es decir, gametos y embriones humanos. Estas directrices son vinculantes para los países firmantes. La justificación de esta reserva se basa en el hecho de que existe una amplia incertidumbre científica sobre los efectos potenciales que las modificaciones genéticas pueden tener en el genoma de futuras personas. Por ello, toda investigación que implique la edición genómica en línea germinal debe cumplir con los artículos 13 y 18 del Convenio de Oviedo que regula la investigación con embriones in vitro y prohíbe la creación de embriones con fines de investigación. La investigación y edición genética con embriones sobrantes de fecundación in vitro está permitida, pero nunca su uso con fines reproductivos^95^.

Los avances y las investigaciones en este campo son progresivos, con el objeto de mejorar su efectividad y con el punto de mira puesto en el mosaicismo, la heterocigosidad, o los errores e imprevistos derivados de la técnica, siendo acordes con la legislación vigente y los principios de la bioética. Se trata de un proceso tan complejo como cuestionado por el potencial de su uso con fines eugenésicos^96^, o ante intervenciones en la línea germinal con consecuencias irreversibles. Sin embargo, la investigación en EGLGH está aún muy lejos de alcanzar la fase de ensayo clínico. Dadas las limitaciones existentes y las restricciones éticas relativas a la EGLGH, se han evaluado modelos alternativos, como las células madre, en términos de su valor predictivo sobre el resultado genético para los enfoques de EGLGH^97^. 

Una de las primeras cuestiones es determinar cuál puede ser el alcance de las transformaciones CRISPR-Cas9 en relación con la seguridad, la eficiencia y los efectos adversos desconocidos. Efectos ocasionados fuera de la diana: las modificaciones que pueden desencadenarse después de la edición de un determinado gen siguen siendo desconocidas. Por otro lado, el mosaicismo en embriones editados presenta un problema crucial, ya que invalidaría la terapia de corrección génica y dificultaría la posibilidad de predecir los resultados de la edición de genes a través del diagnóstico genético preimplantacional (DGP) para aplicaciones clínicas^98^. Además, es indispensable valorar la influencia del componente ambiental sobre el genoma. 

Una segunda cuestión ética sobre la EGLGH es diferenciar los usos terapéuticos de las tecnologías (el uso de tecnologías para la edición del genoma destinadas a tratar o curar una enfermedad) con fines de mejora^99^. 

Una tercera cuestión son las limitaciones una vez logradas la efectividad y seguridad, pues en principio podríamos suponer que irán destinados a corregir trastornos genéticos causados por un gen (monogénicos), no obstante, muchas de las patologías existentes son poligénicas. 

El Informe del Consejo Nuffield sobre Edición del Genoma y Reproducción Humana nos advierte que la EGLGH puede contribuir a una mayor marginación social y discriminación contra las personas que viven con discapacidades, podría reducir la voluntad social de tener en cuenta la planificación de políticas ante esas patologías generando una mayor vulnerabilidad^100^^,^^101^.

### El desarrollo de úteros artificiales

Paralelamente a lo descrito hasta ahora, hace más de cuatro décadas que diversos grupos dirigen sus investigaciones hacia el conocimiento de la implantación intrauterina y el desarrollo de modelos de placentas artificiales^102^^,^^103^. En este contexto, el útero artificial aún no es una realidad. Sin embargo, parece que podría estar cada vez más próximo el desarrollo de tecnologías destinadas a garantizar la evolución de los embarazos ex vivo^104^^,^^105^^,^^106^. La investigación y estudio del desarrollo embrionario y fetal persiguen consolidar el diseño de un útero artificial que, de lograrse, supondría un cambio en la realidad científica y social^107^. Los úteros artificiales conformarían una estructura capaz de albergar y mantener el desarrollo de fetos humanos y mejorar la viabilidad de los embarazos a término^105^. Es un ámbito de investigación muy atractivo para la ciencia y la industria, con grandes expectativas a desarrollar durante el presente siglo^108^^,^^109^. El éxito de estas investigaciones podría dar respuesta a diferentes circunstancias que comprometen la viabilidad de una gestación a término, tales como las derivadas del ámbito de la patología uterina o de otras situaciones clínicas adversas en la mujer gestante, también en los casos de prematuridad calificada como la principal finalidad^110^^,^^111^. De lograrse, la gestación humana fuera del vientre materno será un nuevo hito en la historia de la reproducción asistida. 

No es de extrañar que, aun cuando puede ofrecer múltiples beneficios, surja un debate ético sobre el uso con fines ectogénicos y con ello sobre los fines y los límites del uso de las nuevas tecnologías en salud^112^. 

En el abordaje del útero artificial debemos hacer diferencia entre dos procesos, lo que algunos autores han denominado ectogénesis parcial, calificando el útero artificial como un soporte biotecnológico para el desarrollo fetal cuando la gestación extracorpórea se enfrenta a complicaciones^113^, y la ectogestación, en la cual un embrión procedente de fecundación in vitro, sería implando y desarrollado a término de forma extracorpórea^114^.

Uno de los dilemas éticos es el que plantea un escenario en el cual el uso de esta tecnología es meramente social o profesional, nos referimos a razones puramente estéticas o de carrera profesional, o como han sugerido algunos autores, encaminadas a una industrialización de la gestación^104^.

Una técnica diseñada y destinada a preservar y mejorar la vida y la salud de fetos prematuros puede también albergar profundos riegos bioéticos^4^^,^^115^^,^^116^. Podría ser apropiado que la legislación llegase a regular los derechos y obligaciones de los pacientes, equilibrando una posible delimitación de la autonomía reproductiva de los progenitores^117^. Amparado en el prinipio de igualdad y justicia distributiva, la ectogestación podría permitir el acceso a la paternidad de varones solos o parejas homosexuales como alternativa (no sin importantes dilemas) a la gestación subrogada. Parece que la ectogestación sería un paso hacia la igualdad; sin embargo, surgen planteamientos contrarios que cuestionan si desencadenaría una restricción en las libertades individuales de la mujeres sobre la gestación y su propio cuerpo, incluso llegando a plantar, en determinadas culturas, la anulación de algunos derechos de la mujer por las decisiones del conyuge o de otras mujeres de la familia^118^^,^^119^.

Llegamos al tercero de los planteamientos, en el que se pueden abordar los conceptos de maternidad y paternidad en una gestación extracorpórea y la potestad adquirida en los eventuales sucesos que pueden derivarse, como sería el caso de la interrupción de la gestación, en particular ante una ectogénesis total^112^. Afectaría de igual modo a la Ley 14/2006 de Reproducción humana asistida, en relación con los límites temporales en el desarrollo embrionario *in vitro*^36^^,^^38^^,^^40^, o en relación con la premoriencia de los progenitores. También afectaría a la Ley 14/2007 de Investigación biomédica y la Ley 2/2010 de Salud sexual y reproductiva en materia de interrupción voluntaria de la gestación^120^^,^^121^. La nueva posibilidad de ectogestación reavivaría el debate sobre el estatus jurídico del embrión y del feto humano, o incluso requeriría de un debate normativo sobre una nueva condición de ser vivo durante el desarrollo extracorpóreo. Deben estudiarse y valorarse también los riesgos y responsabilidades en el uso o indicación de las nuevas biotecnologías invasivas, tales como anomalías físicas o morfológicas como a nivel del deterioro del bienestar fetal, más allá del proceso fisiológico. Además, sería interesante estudiar un marco normativo seguro ante acciones ilícitas con los recién nacidos; en definitiva, abordar nuevamente viejos planteamientos clásicos propios del ámbito de la biología reproductiva, como el inicio de la vida, el concepto de viabilidad fetal y las consecuentes repercusiones éticas y jurídicas de todo ello. 

## CONCLUSIONES

Es incuestionable que el incesante desarrollo tecnológico ha generado un sinfín de posibilidades en el ámbito de la biomedicina y, en particular, en la biología reproductiva. Así, los nuevos avances traen consigo un cambio en la actitud y los posicionamientos respecto al inicio de la vida que requieren de un diálogo entre el Derecho y la Ciencia tan necesario como complejo y en el que la bioética debe estar más presente que nunca. Las leyes que regularán dichos avances son producto de las sociedades, de los valores y de las épocas, tal y como explica Gracia: “Dime qué valores tiene una sociedad y te diré qué leyes hay”^43^.

Es inevitable que las nuevas tecnologías lleguen al ámbito de aplicación clínica y, en este escenario, suele ocurrir que el desarrollo científico avanza más rápido que su regulación jurídica, corriendo el riesgo de que un uso irresponsable de los avances en ciencia y tecnología generen riegos para el ser humano^8^^,^^122^. 

Con relación a los avances analizados en este trabajo, la criopreservación de ovocitos es una técnica que persigue paliar el retraso de la edad de maternidad; sin embargo, no es una solución a ella, debiendo abordase desde una perspectiva social integral. 

La problemática de los embriones sobrantes sigue presente en muchos países, sin que sean eficaces las medidas jurídicas sobre su uso, custodia y destino establecidas. Entre los avances más recientes, el trasplante uterino y la investigación para el desarrollo de úteros artificiales abre la puerta a debatir sobre la ectogestación. Ambos procedimientos buscan contribuir al tratamiento de la infertilidad y la mejora del pronóstico en partos prematuros; sin embargo, deben perfeccionarse, atender a los condicionantes éticos que hemos descrito y ser regulados jurídicamente.

El uso de técnicas complementarias a los tratamientos de fecundación in vitro plantea un auténtico desafío en la autoregulación de la ciencia y la medicina reproductiva, siendo necesario conocer el posicionamiento de las asociaciones científicas sobre la aplicación de técnicas en desarrollo, experimentales, o con una evidencia aún por determinar, de modo que supone a su vez un reto en materia de información, consentimiento, implicaciones económicas y resultados.

Para concluir, la edición genética y la reprogramación celular siguen siendo dos grandes promesas de la investigación en biología reproductiva y genética humana con complejas aristas ético-jurídicas aún por abordar. En resumen, el futuro plantea la necesidad de prestar especial atención a los retos presentes y futuros, pues los fines biomédicos y reproductivos pueden verse redirigidos hacia algo más que curar.
